# Keyword Index for Volume 100

**DOI:** 10.1038/sj.bjc.6605133

**Published:** 2009-06-09

^1^H MRS 789

17*α*-hydroxylase/C17,20-lyase 671

17-AAG 334

2-methoxyoestradiol 476

25(OH)D 450

5-fluorouracil 246, 881, 1647

8-oxo-dG 381

abiraterone 671

absolute risk increase 913

absolute risk reduction 913

accelerated/dose-dense chemotherapy 305

active surveillance 888

acute lymphoblastic leukaemia 1026

acute myeloid leukaemia 1026

acyl-CoA synthetase 1369

acylpeptide hydrolase (ACPH, EC 3.4.19.1) 723

ADCC 113

adenocarcinoma 1400

adenoma 1674

adiposity 578

adjuvant chemotherapy 732

adjuvant radiotherapy 913

adjuvant therapy 1549

adoptive Th1-cell therapy 1135

advanced colorectal cancer 266

advanced stage 1198

adverse effects 1

Africa 799

age at first birth 538, 1832

agent-based model 1917

AKT 649, 782, 1406, 1523

Akt/PKB activation 932

ALA derivatives 723

allelic imbalance 1336

alternative splicing 228

aminolaevulinic acid 723

AML 1343

amosite 1175

amplification 487

anal cancer 693

androgen independent 1784

androgen synthesis 671

aneuploidy 959

angiogenesis inhibitors 1, 865, 971, 1465, 1575, 1617, 1666

animal models 221, 311, 1755

anogenital cancers 527

antibiotics 834

antibody-dependent cellular phagocytosis (ADCP) 113

anti-neoplastic-combined chemotherapy protocols 44

anti-tumour strategy 1755

ApoE: apolipoprotein E 1966

Apollon 739

apoptosis 106, 118, 322, 381, 590, 739, 789, 923, 1068, 1073, 1154, 1347, 1415, 1879

AQP3 1889

aromatase inhibitors 442

arteriogenesis 865

asbestos exposure 1336

Ashkenazi 583

aspirin 551

association studies 993, 1846

asthma 829

astrocytoma 789

ATP7A 96

AU-rich element 1943

Aurora A 959

autocrine 971

autoimmune diseases 817, 822

autophagy 1154

*β*-catenin 1292

BAG-1 123, 1347

basal 405

basal cell 174

Bcl-2 family 1896

BCL6 1320

BCR-ABL 1523

BCRP 1120

bevacizumab 1111, 1704, 1842

bilateral breast cancer 563

biliary tract neoplasms 194

biobanks 8

biological markers 1608

biomarkers 1, 145, 888, 1219, 1534, 1603

birth length 185, 803, 1794

birth weight 185, 803, 1794

bladder cancer 834

bladder infection 834

bleomycin 464

body composition 1799

body fat distribution 1486, 1799

BOLD 644

bone cancer 188

bone marrow 153, 1937

borderline ovarian tumour 1731

bortezomib 366, 1379

brachyury 1406

*BRAF* 431, 1087

brain metastases 291, 894

brain tumour 185, 1154

*BRCA1* 421, 583

*BRCA2* 421, 583

breast cancer 123, 281, 376, 405, 442, 494, 545, 578, 583, 590, 598, 633, 680, 739, 764, 807, 811, 817, 918, 959, 1043, 1055, 1205, 1277, 1465, 1508, 1687, 1746, 1794, 1806, 1873, 1879

breast cancer histology 538

breast cancer resistance protein 476

breast cancer risk 1492

breast carcinoma 1048

breast density 1205

breast diseases 1873

breast neoplasm 77, 1486

breast-conserving surgery 1048

C6-pyridinium ceramide 626

CA IX 874

CA125 1315

cachexia 63, 713

cancer incidence 167, 170, 188, 206, 266, 545, 853

cancer pain 1566

cancer registry 799

cancer risks 421, 829, 1021

cancer stem cells 221, 1917

cancer survival 858

cancer-linked hypomethylation 389

capecitabine 37, 1704, 1842

carbogen 644

carbonic anhydrase 405

carboplatin 50, 707

cardiac 684

cardiovascular toxicity 1861

carotenoid 181

case–control studies 200, 558, 795, 1175, 1483, 1812

caspase-3 739

castration-refractory prostate cancer 671

CBF1 1957

CD133 950

CD44 918

CD45 366

*CDA*208G>A 870

*CEBPA* 1343

cell cycle 1957

cell cycle arrest 1879

cell invasion 633

cell-cycle proteins 1128

CellSearch 160

central nervous system tumour 185

cerebral infarction 811

cerebrovascular 811

cervical cancer 532, 1191, 1617, 1832

cervical carcinoma 1303

cervical intraepithelial neoplasia 1184

cetuximab 251, 298, 950, 1032, 1379, 1704

CHEK2 1508

chemical genetic interactions 1213

chemical synthetic lethality 1213

chemoimmunotherapy 28

chemokine receptor 1444, 1755, 1949

chemoprevention 281

chemoradiation 246

chemoradiotherapy 37

chemoresistance 1647

chemotherapy 50, 82, 266, 298, 311, 431, 455, 874, 881, 1032, 1144, 1287, 1842, 1896

child sexual abuse 1191

childhood cancer 82, 188, 213

childhood exposure 1021

China 532

Chk1 1425

cholangiocarcinoma 178, 1257, 1765

cholecystitis 194

chordoma 1406

chromosomal instability 1517

chronic myeloproliferative diseases 822

CI-1040 370

cigarette smoking 1483

ciprofloxacin 1581

circulating DNA 1277

circulating tumour cells 153, 160, 1277

c-Jun 1415

clinical benefit 1373

clinical management 1731

clinical outcome 511

clinical trial, phase II 44

clomiphene 1824

cluster analysis 1575

CMET 145

CNS PNET 1292

cohort effects 527

cohort studies 551, 1503, 1799

colon and gastric cancers 663

colon cancer 381, 611, 1508, 1755

colon carcinoma 381, 1415

colorectal adenoma 1812

colorectal cancer (CRC) 56, 233, 251, 360, 511, 676, 701, 803, 881, 1087, 1095, 1236, 1330, 1452, 1530, 1534, 1540, 1549, 1659, 1674, 1704, 1966

colorectal cancer screening 1103

colorectal liver metastases 617

colorectal neoplasms 259, 1230

combination chemotherapy 315, 601, 979

combination therapy 1647

combination treatment 1896

combined hepatocellular cholangiocarcinoma 1765

complex I deficiency 1434

complex intervention 274

continuity of care 274

copper and neuroblastoma 96

core needle biopsy 1771

cosmesis 1680

cost-effectiveness 70, 281, 598, 1240

Cox regression 1393

coxsackie adenovirus receptor 352

CpG island hypermethylation 663, 1534

CPT-11 1581

CRM1 1943

CRP gene 1846

cryotherapy 1889, 1896

curative resection 701

curcumin 1425

cut-off 259, 1103

CXCL-8 1638

CXCR1 1638

CXCR2 1638

CXCR4 1949

cyclin B1 1055

cyclin D1 1292

cyclin D2 1320

cyclin-dependent kinase (CDK) 494

CYP3A4 412

CYP450c17 671

cytochrome *c* 1912

cytokines 1617

database study 178

DCE-MRI 1575

decision making 590, 913

decisional conflict 590

decitabine 758

deleterious mutation 426

dendritic cells 1111

detection 160

developing countries 858, 1026

diabetes mellitus 795

diagnosis 56, 908, 1731, 1873

diagnostic errors 1873

dietary glycemic load 558

diffuse-type gastric cancer 389

dihydroceramide 626

disseminated 160

distant recurrence prediction 494

DLEC1 663

DNA 399

DNA copy number analysis 1517

DNA damage 1425

DNA methylation 240, 344, 758, 1534

DNA replication licensing 959

docetaxel 436

dose finding 1739

downregulation 1095

doxorubicin 305

DR4 1415

DR5 1415

drainage 464

drug interaction 315

drug resistance 979, 1120, 1144, 1517, 1926

drug sensitivity 758

drug treatment 1267

DU145 649

ductal carcinoma *in situ* 1048

dysplasia 1128

E2F-5 764

E-7107 228

early breast cancer 305, 563

early diagnosis 1867

echocardiography 1861

economic evaluation 1240

EGF 334, 1358

EGFR pathway 19, 89, 145, 1257, 1379, 1523

ELISA 1659

endometrial carcinoma 89, 913, 1358

endometriosis 1315

enterolignans 1492

ependymoma 185

EphA1 1095

epidemiology 167, 200, 206, 807, 840

epidermal growth factor receptor 298, 623, 732, 950, 1087

epigenetic silencing 1534

epigenetic therapy 28

epigenetics 240, 344, 571, 1687

epirubicin 305

epithelial ovarian carcinoma 1144

epithelial solid tumours 1379

epithelial–mesenchymal transition 134, 389

epithelium 221

*ERBB2* 487

ErbB2/Her-2 633

erlotinib 1120

ESR1 1358

ethics 8

ethnicity 545

everolimus 315

evidence synthesis 1219

Ewing sarcoma 188

excision repair cross-complementing gene 1 732

exemestane 442

export 1943

extra-cellular 1287

extracellular matrix 1589

extracranial disease control 894

faecal occult blood test 259

faeces/chemistry 1230

family history 524

*FANCJ/BRIP1* 426

fatty-acid synthase 1369

feasibility trial 274

febrile neutropenia 436

female survivors 77

fibrosis 1680

FIGO stage I–IIB 1400

Filipino-Americans 858

first sexual intercourse 1191

first-line treatment 251, 1720

fish oil 713

fluorescence *in situ* hybridisation 487

fluorescence-activated cell sorting 1452

fluoropyrimidine 251

fluorouracil 1549

focal adhesion 633

folate 233, 1120

follow-up 70, 1852

fotemustine 322

Frizzled 1165

fruits 181

*γ*-secretase 1879

*γ*-secretase inhibitor 1957

G2/M arrest 1425

G67E 376

gall bladder cancer 178

gallbladder neoplasms 194

gallstones 194

gastric adenocarcinoma 1483

gastric cancer 153, 298, 352, 487, 551, 558, 732, 772, 782, 1320, 1937

gastric pit cell 389

gefitinib 1120

gemcitabine 37, 246, 601, 870, 1032, 1842

geminin protein 1128

gender differences 799

gene expression 511, 1517, 1926

gene expression microarray analysis 1452

gene silencing 1534

gene-expression profiling 656

general practice 24

genetic counselling 583

genetic marker 1336

genetic risk factors 1471

genetic screening 1777

genetic susceptibility 412

genetic synthetic lethality 1213

genistein 1817

genomic 1471, 1517

GEP NETs 501

glasgow prognostic score 701, 1236

glioblastoma 106

glioblastoma multiforme 950

glioma 1154

glucocorticoids 1926

glycemic index 558

gonadal function 455

gonadotrophins 1824

guaiac 1230

guaiac-based faecal occult blood test 1103

guidelines 1219, 1852

gut 1581

gynaecological cancer 1558

HAART 840

haematopoietic malignancies 113, 524

HB-EGF-CTF 1320

HE4 1315

heat-shock proteins 1523

hedgehog 389

*Helicobacter* 194

helper epitope 1135

hepatic progenitor cells 1765

hepatitis viruses 840

hepatocellular carcinoma 181, 799, 1385, 1647, 1765

HER2 680, 764, 1061, 1523

HER-2/neu 89

HER2-overexpressing breast cancer 894

Her/ErbB receptor family 941

heterogeneous nuclear ribonucleoprotein K 1608

HIF-1*α* 772

HIF-1*α* and HIF-2*α* 1666

histamine-2-antagonists 1503

histone deacetylase inhibitor 28, 758

histone marks 240

HIV 840

HMGA1 501

HMGA2 501

*hMLH1* 376

HNPCC 376

hormone receptor status 807

hormone refractory prostate cancer 13

HOX 470

HSC70 1347

HSP70 1347

Hsp90 334

HT-29 1575

human immunodeficiency virus/acquired immunodeficiency syndrome (HIV/AIDS) 799

human papillomavirus 527, 532, 840, 1184

humans 1452

HuR 1943

hypermethylation 1438

hypoxia 405, 644, 772, 1666

hypoxia-inducible factor-1 747, 1444

*ID1* 932, 1937

IGF-1 334, 1794

IGF-1/IGF-1R 366

IGF-2 1794

imatinib 923

immunity 1581

immunochemical faecal occult blood test 1103

immunochemical test 259

immunocytology 1627

immunohistochemistry 1608

immunology 1061, 1846

immunotherapy 1697, 1746

*in utero* exposure 213

*in vivo* imaging 1257

incidental findings 8

incidental prostate cancer 170

India 848

infectious complications 1236, 1581

infertility 1824

inflammation 63, 1846

information 590

informed consent 8

inhibin-*α* subunit 1784

in-patients 908

INRG 1471, 1627

insulin-like growth factor 1794

Int7G24A 1674

interferon-*α* 1647

international consensus 1471

inter-observer 901

intervention 590

intraductal papillary mucinous neoplasm of the pancreas 1438

intrapericardial instillation 464

intra-tumoural 1287

invasion 352, 1638

ionising radiation 213, 1021

irinotecan 50, 881, 1704

isoflavones 649, 1812, 1817

Janus kinase 2 134

Japan 1812

JNK1*α*1 1415

kallikrein-related peptidases 1659

Ki-67 888

KLKs 1659

*K-Ras* 145, 370, 1087

*K-Ras* mutations 656, 985

*Kras* overexpression 656

lapatinib 89

larynx cancer 167

late side effects 1680

late toxicity 1558

left truncation 1806

leptin 578

*let-7* 501

leucine 713

leukaemia 524

lignan 1817

lipid metabolism 1369

lipopolysaccharide 1589

liver metastasis 1540

liver resection 617

LKB1 370

LLL-3 106

LNcaP 1068

lomeguatrib 1245, 1250

longitudinal study 1558

Los Angeles 834

lung cancer 56, 167, 291, 464, 470, 941, 1037, 1336, 1896, 1949

lymph node metastasis 1937

lymphoid tumours 1926

lymphoma 524, 1771

macrophages 113

magic angle spinning (MAS) 789

magnetic resonance imaging 644

male lymphoma survivors 455

malignant neoplasms 1205

malignant pericardial effusion 464

malignant spinal cord compression 1867

mammography 901, 1205, 1486

markers 221

mass screening 1230

massARRAY 344

mastectomy 1048

maximal tolerated dose 1373

Mcm2 protein 1128

mean sojourn time 1198

medical oncology 44

medulloblastoma 1292

MEK 370

melanoma 28, 174, 431, 1245, 1250, 1638

melanoma therapy 322

menarche 538

menopause 538

mental health 908

mesothelin 1144

mesothelioma 1175

Met 941

meta-analysis 436, 551, 611

metabonomics 923

metachronous breast cancer 563

metastases 153, 251, 633, 772, 1073, 1444, 1755, 1784, 1842

metastatic colorectal cancer 1720

methotrexate 979

methylation 399, 571, 663, 1095, 1277

MGMT 322

micrometastases 160, 360

microRNA 1002, 1517

microvessel density 1666

migration 134, 352, 1917

minimal disease 1627

minor surgery 24

mismatch repair 266, 322

mitochondria 381

mitochondrial dysfunction 1912

MLH1 758

MMR: mismatch repair 1966

molecular analysis 1771

molecular imaging 747

molecular marker 360, 1330

molecular therapy 19

molecularly targeted agents 1373

molecular-targeted therapy 1257

monitoring 684

mortality 170, 206, 450, 853, 1205, 1824, 1837

MSI-H: high-frequency microsatellite instability 1966

MSS/L: microsatellite stable/low-frequency microsatellite instability 1966

mTOR 431, 782, 971, 1267, 1406

*MUC4* 344

mucin 344

mucinous 881

multicentre survey 1731

multidrug resistance 476

multiple myeloma 366

murine fibroblast cell lines 656

muscle 713

mutation screening 623

mutations 1330, 1343

myelodysplastic syndrome 822

myeloid leukaemia 822

nasopharyngeal carcinoma 1002

national databases 829, 1499

neoadjuvant therapy 442

neoplasm circulating cells 608

neoplasms 44, 908

neuroblastoma 399, 853, 1471, 1627

nimotuzumab 950

non-Hodgkin’s lymphoma 113, 200, 524

non-progression rate 1373

non-small cell lung cancer (NSCLC) 145, 370, 470, 985

non-steroidal anti-inflammatory drugs 178

Notch 1879, 1957

novel therapeutics 13

NSAIDs 551

nuclear magnetic resonance 923

nuclear matrix 1608

nutrition 713

*O*^6^-methylguanine-DNA methyltransferase 1245, 1250

occult blood 1230

occupational exposure 213

oesophageal adenocarcinoma 1483, 1725

oesophageal cancer 334, 551

oesophageal cancer surgery 70

oesophageal neoplasms 795

oestrogen metabolism 412

oestrogen receptor 764

oestrogen replacement therapy 1486

older women 1043

oncocytic thyroid tumour 1434

oncogenes 575, 993, 1213

oncolytic adenovirus 1154

opioids 1566

OPN 1746

oral cancer 848, 1128, 1943

oral glucocorticoids 200

osteosarcoma 188, 1957

ovarian carcinoma 134, 412, 476, 601, 707, 971, 1315

ovarian neoplasms 1

ovarian stimulation 1824

overdiagnosis 1198

oxaliplatin 251, 601, 881, 1032, 1704

oxidative damage 381

P2Y 1465

p53 739

p73 1347

paclitaxel 315, 707

Palomid 529 932

pancreatic adenocarcinoma 1444

pancreatic cancer 37, 246, 870, 1032, 1267, 1425, 1842

parity 538

passive smoking 1483

pathological complete response 1725

pathway 1002

pathway biomarker 1393

patient satisfaction 70

PBX 470

PDT 626

perceived risk 583

perinatal 803

period analysis 858

perioperative metastatic tumour growth 1589

peripheral blood 153, 1937

peritoneal dissemination 1937

persistence 1184

persistent trophoblastic disease 979

PET CT 693

Petersen Index 701

pH 1287

pharmacogenetics 732, 1549

pharmacogenomics 870

pharmacokinetics 1549

Phase-I trial 315, 1373

Philippines 858

phosphotyrosine 1465

photodynamic therapy 723

Photofrin 626

physical activity 611

PI3K 431, 1267

PK/PD 1739

planiedolide 228

plant lignans 1492

plasma 626, 1817

platinum-resistant 601

PMI 1566

PNA 985

polychotomous logistic regression 1483

polymorphism 870, 993, 1358

pooled-analyses 412

population-based 853, 1103

portal vein embolisation 617

positive lymph node ratio 1530

preconception irradiation 213

predictive biomarkers 405, 1393

predictive factor 874

predictive testing 959

pregnancy 1191

preoperative chemotherapy 1725

prevalent cases 1806

prevention 233

primary hormonal therapy 442

primary liver cancer 799

primary studies 1219

primary xenograft 1267

privacy 8

prognosis 123, 145, 266, 352, 450, 676, 874, 959, 1055, 1219, 1343, 1385, 1486, 1540, 1777

prognostic marker 1534

prognostic tumour marker 1219

prolyl hydroxylase 1687

promiscuous peptide 1135

prophylaxis 281

prospective 1817, 1861

prospective study 181

prostasomes 1603

prostate 376, 649

prostate cancer 56, 240, 450, 644, 888, 932, 1068, 1165, 1198, 1508, 1603, 1608, 1784, 1817, 1846, 1852, 1889

prostate cancer predisposition 426

prostatic neoplasms 608, 1799

proteasome 1879

proteasome inhibition 1379

proteomics 1303

proton pump inhibitors 1503

protoporphyrin IX 723

PSA biomarker 13

psoriasis 1499

PTEN 431, 1087

public trust 8

purinergic receptor 1465

QRT-PCR 1627

quality 1566

quality of life 70, 82, 311, 1558

quantitative PCR 1095

RAD001 315

radiation 206, 334, 950

radiation therapy 747

radiation-induced bystander effect 1912

radical hysterectomy 1400

radiochemotherapy 291

radiographer 901

radiologist 901

radioresistance 932

radiotherapy 693, 811, 874, 1048, 1558, 1680

raloxifene 281

randomised clinical trial 1037

randomised trial 1103

randomized controlled trial 44

randomized trial 246

rapamycin 1406, 1739

rapid referral system 1867

*RASSF1A* 399

real-time PCR 985

receptor tyrosine kinase inhibitors 19

recommendations 684

rectal cancer 874, 1666

relapse 598, 707

renal cell carcinoma 1111

resistance 707

respiratory chain 1434

response 1287

response marker 123

*RET* proto-oncogene 1777

retinoic acid receptor *β*_2_ 96

retinoids 96

retropubic prostatectomy 608

reversibility 118

rheumatic diseases 817

risk 1184, 1503

risk assessment 1343

risk factors 178, 185

risk of ovarian cancer 993

RNAi 1213

RTK 941

RUNX3 676

salivary gland carcinomas 623

SCLC 1949

sclerosis 464

screening 259, 532, 901, 1043, 1198, 1205, 1240, 1832

seasonal variation 185

second primary cancer 77

self-metastases 1917

sensitivity 985

sensitivity and specificity 908, 1230

serum 399, 450

sexual behaviour 1191

sFRP1 1165

signal transducer and activator of transcription 3 134

signal transduction 649, 941, 1949

SIMS 63

single-agent chemotherapy 979

siRNA 739

siRNA silencing 723

skin cancer 174, 200

skin malignancy 24

SMAD4 1438

small RNAs 240

SNP: single nucleotide polymorphism 1966

SNPs 426

somatic mutation 1777

sorafenib 19, 1111

spliceosome 228

spliceostatin A 228

sPNET 1292

sporadic medullary thyroid carcinoma 1777

squamous cell 174

stage 545, 693, 1530

STAT3 106, 1949

stem cells 221, 240

STn 1746

stomach neoplasms 1503

stroke 811

SU6668 1575

sulphamate 476

sunitinib 1111

surgery 56, 1236

surgery prognosis 608

survival 118, 188, 381, 545, 563, 701, 807, 853, 1055, 1358, 1385

survival analysis 1806

survivin 1073

synchronous breast cancer 563

syngeneic tumour 1287

systematic review 436, 1852

tamoxifen 281, 1048

tamoxifen sensitivity 123

target 1002

targeted therapy 431, 623, 1330

TATI 1540

telangiectasia 1680

temozolomide 322, 1154, 1245, 1250

testicular cancer 1861

TGFBR1 1674

TGFBR1^*^6A 1674

Th17 1061

therapeutic antibodies 113

therapy 1731

Theratope 1746

thymidine phosphorylase 732

tissue arrays 676

tissue microarray 511

tobacco chewing 848

Toll-like receptor 4 1589

topotecan 291

toxicity 601, 870

*TP53* 1330, 1680

TRAIL 1415

transcription 1347

transcription factor 511, 764

transforming growth factor-*α* 298

translation factors 1393

translational 1471

translocation (4,14) 366

transurethral resection of the prostate (TURP) 170

trastuzumab 89, 680, 684, 894, 1061

treatment 545

treatment planning 1471

treatment-related death 1026

T-regulatory 1061, 1697

trends 77, 167

trial effect 1037

trial participation 1037

triple drug combination 1720

tumour 118, 626, 789

tumour blood vessels 865

tumour exosomes 1603

tumour hypoxia 747

tumour markers 1659

tumour microenvironment 1073

tumour progression 1917

tumour suppressor genes (TSG) 663, 1213, 1523

tumourigenesis 1638

tumour-specific CD4^+^ T cells 1135

UK 684

uninformative results 421

unknown primary 44, 50

urinary tract infection 834

urokinase plasminogen activator system 1589

urokinase-type plasminogen activator receptor (*uPAR*) 153

uterine cervix 1400

vaginal carcinoma 1303

valproic acid 28

vascular endothelial growth factor 1, 1385

vascular heterogeneity 1575

vascular targeting agent 19

vascular thromboembolism 1837

vegetables 181

VEGF 932, 1111, 1257, 1465, 1523

VEGF-A 865, 971

VEGFR2 971, 1465

venogenesis 865

viral hepatitis 1765

vitamin C 181

wasting 713

watchful waiting 888

weekly 707

western blot 1608

WNT pathway 1292

Wnt signalling 676, 1165, 1647

Wnt5a 1165

workers 206

WW domain-containing oxidoreductase 1438

XELOXIRI 1720

xenografts 13, 918

